# Genomic Characterization of *mcr-1-*carrying *Salmonella enterica* Serovar 4,[5],12:i:- ST 34 Clone Isolated From Pigs in China

**DOI:** 10.3389/fbioe.2020.00663

**Published:** 2020-06-30

**Authors:** Mohammed Elbediwi, Beibei Wu, Hang Pan, Zenghai Jiang, Silpak Biswas, Yan Li, Min Yue

**Affiliations:** ^1^Institute of Preventive Veterinary Sciences, Department of Veterinary Medicine, College of Animal Sciences, Zhejiang University, Hangzhou, China; ^2^Zhejiang Province Center for Disease Control and Prevention, Hangzhou, China; ^3^College of Veterinary Medicine, Henan University of Animal Husbandry and Economy, Zhengzhou, China; ^4^Zhejiang Provincial Key Laboratory of Preventive Veterinary Medicine, College of Animal Sciences, Zhejiang University, Hangzhou, China

**Keywords:** *Salmonella enterica* serovar 4, [5], 12:i:-, ST34, Colistin, *mcr-1*, food chain

## Abstract

*Salmonella enterica* serovar 4,[5],12:i:-, so-called Typhimurium monophasic variant, has become one of the most frequently isolated serovars both in humans and in animals all over the world. The increasing prevalence of *mcr*-1-carrying *Salmonella* poses significant global health concerns. However, the potential role of *Salmonella* 4,[5],12:i:- in *mcr*-*1* gene migration through the food chain to the human remains obscure. Here, we investigated 337 *Salmonella* isolates from apparently healthy finishing pigs, which is rarely studied, obtained from pig farms and slaughterhouses in China. The *mcr*-*1* gene was found in four colistin-resistant *S. enterica* 4,[5],12:i:- isolates. Notably, all four isolates belonged to sequence type 34 (ST34) with multidrug resistance phenotype. Further genomic sequencing and antimicrobial resistance characterization confirmed that *mcr* was responsible for the colistin resistance, and the conjugation assay demonstrated that three of four isolates carried *mcr*-*1* in IncHI2 plasmid. Importantly, *mcr*-*1* and class-1 integron were found to co-localize in two strains with IncHI2 plasmid. By collecting all the *mcr*-*1*-carrying Typhimurium and monophasic variant strains across the food chain (farm animals, animal-origin food, and humans), our phylogenomic analysis of available 66 genomes, including four strains in this study, demonstrated an independent phylogenetic cluster of all eight Chinese swine-originated isolates and one human isolate. Together, this study provides direct evidence for clonal and pork-borne transmission of *mcr-1* by *Salmonella* 4,[5],12:i:- ST34 in China and highlighted a domestication pathway by acquisition of additional antimicrobial resistance determinants in Chinese ST34 isolates.

## Introduction

*Salmonella* spp. are important zoonotic pathogen commonly identified in farmed livestock. A particular monophasic variant of *Salmonella* Typhimurium (*Salmonella* 4,[5],12:i:-) emerged in the mid-1990s and became one of the most widespread serovars (Hopkins et al., 2010; Wang et al., 2019). Recently, some studies proposed it as the global pandemic clone (Alicia et al., 2018; Mulvey et al., 2018; Biswas et al., 2019). Importantly, *Salmonella* 4,[5],12:i:- were suggested to be associated with swine (Linxian et al., 2017) and currently considered with high frequency of multidrug resistance potential among *Salmonella* serovars, posing significant public health concerns worldwide (Elnekave et al., 2018; Mather et al., 2018; Monte et al., 2019).

*Salmonella* serovar 4,[5],12:i:- is also commonly found in isolates from humans in China and has been linked to food animals and animal-borne products, particularly swine and pork (Linxian et al., 2017; Paudyal et al., 2018). Pork products are considered as one of the main sources of human S*almonella* infections (Lu et al., 2019).

As a result of the extensive use of colistin for veterinary purposes especially for the control and prophylaxis of *Enterobacteriaceae* infections, the mobilized colistin-resistant (*mcr*) *Salmonella* 4,[5],12:i:- has been disseminated in humans, animals, as well as food products, including pork (Linxian et al., 2017; Alicia et al., 2018; Elbediwi et al., 2019). *mcr* carrying *Salmonella* 4,[5],12:i:- has also been reported from Asia, European countries, and North America (Monte et al., 2019). However, the study of *Salmonella* 4,[5],12:i:- in the finishing pigs is largely lacking and the direct evidence of the pork-borne transmission, particularly in certain critical antimicrobial resistance, i.e., mobile colistin resistance gene migration, remains obscure in China. Here, by focusing on the asymptomatic finishing pigs, an underappreciated modulator in the pork-borne transmission chain, we aimed to characterize the genomic features and evaluated the potential role of pork-borne transmission for *mcr-1*-carrying *Salmonella* 4,[5],12:i:-.

## Materials and Methods

### Sample Collection

Randomized sampling was done as a part of epidemiological surveillance for detection of colistin-resistant S*almonella* isolates in finishing pigs. Between March 2017 and November 2017, a total of 1732 fecal samples were collected from randomly selected 45 pig farms, and two pig slaughtering facilities at Henan province in China.

### Isolation and Identification of *Salmonella* Isolates

The isolation, identification, and serotyping of the organisms were done according to previous protocols (Jiang et al., 2019). The obtained isolates were then confirmed as *Salmonella* by polymerase chain reaction (PCR) using specific primers for amplification of the enterotoxin *stn* gene as recommended (Zhu et al., 2015). Monophasic *S.* Typhimurium isolates were serotyped by O-, H- antigens (Jiang et al., 2019) and confirmed by PCR (Bugarel et al., 2012), as described previously.

### Antimicrobial Susceptibility Testing

Broth micro-dilution method was used to determine the minimum inhibitory concentrations (MICs) of 16 antimicrobial drugs for all isolates and results were interpreted according to CLSI protocols (Chattopadhyay et al., 2013). The antimicrobials used include colistin, ampicillin, amoxicillin–clavulanic acid, chloramphenicol, streptomycin, florfenicol, tetracycline, kanamycin, doxycycline, gentamicin, sulfamethoxazole, sulfamethoxazole/trimethoprim, ciprofloxacin, enrofloxacin, ceftriaxone, and cefotaxime.

### PCR Screening of *mcr* Genes

The genomic DNA was extracted from the isolates and subjected for screening *mcr* genes of various types (*mcr-1* to *mcr-8*), using a multiplex PCR as recommended (Wang et al., 2018). To elaborate, PCR amplification was performed using iTaq TM DNA polymerase with the following cycling conditions: 34 cycles of 94°C for 20 s, 50°C for 20 s, and 72°C for 30 s, followed by 1 cycle of 72°C for 5 min.

### Bacterial Conjugation Assay

Bacterial conjugation for the *mcr-1* positive *Salmonella* isolates was done in a liquid and solid mating-out assay (Lampkowska et al., 2008) using *Escherichia coli* J53 (streptomycin- and rifampicin-resistant) to detect the transferability of the gene. Trans-conjugants were selected on LB agar plates containing rifampicin (100 mg/L) and colistin (2 mg/L). The conjugation efficiency rate was estimated as a number of trans-conjugants per total recipients.

### Genomic Sequencing and Data Analysis

The genomic DNA was first extracted and then sequenced on the Illumina MiSeq platform. The quality of sequencing and trimming was checked with FastQC toolkit (Bolger et al., 2014). The raw reads for each strain were assembled by using SPAdes 4.0.1. PLACNETw (Vielva et al., 2017) web tool was used to reconstruct the plasmid genome from the whole-genome sequence. The reconstructed plasmid contigs were aligned against the non-redundant database^1^ to find the best plasmid match. QUAST (Gurevich et al., 2013) was used to assess the assembled genomes through basic statistics generation, including the total number of contig, the length of contig, and N50. Prokka 1.14, with the “default” settings, was used to annotate the assembled genomes. The Genomic DNA library was constructed using Nextera XT DNA library construction kit (Illumina, United States, No. FC-131-1024), followed by genomic sequencing using Miseq Reagent Kit v2 300cycle kit (Illumina, United States, No. MS-102-2002). High-throughput genome sequencing was accomplished by the Illumina Miseq sequencing platform, as previously described (Paudyal et al., 2019; Biswas et al., 2020; Yu et al., 2020).

*Salmonella* monophasic *in silico* serotyping was done by SISTR (Yoshida et al., 2016) web tool. Multilocus sequence typing (MLST), detection of resistance genes, and plasmid replicon were conducted in the Center for Genomic Epidemiology (CGE)^2^ platform. Blastn^3^ and BRIG (Alikhan et al., 2011) tools were used to separate the plasmid contigs of the *mcr-1*-carrying plasmid and display circular comparison. Snapgene software^4^ was used to display the full annotated versions of the *mcr-1-*carrying plasmids (Supplementary Figures S1–S3). Pan-genome analysis was performed using the Roary pipeline (version 3.4.2) (Page et al., 2015).

Phylogenomic analysis was used Snippy v4.4.4 to obtain SNPs alignment and the phylogenetic tree was built by the maximum likelihood method with RAxML^5^ based on the recombination-free SNPs. To identify the phylogenetic relationship of *mcr-1*-carrying isolates, four strains of swine origin isolated in this study and all available *mcr-1* positive *S*. Typhimurium and monophasic Typhimurium genomic sequences were retrieved from GenBank, including two environmental, 14 from swine (live pigs and pork), 17 from other animals, and 31 from humans, and were pooled together for further phylogenetic analysis.

## Results

### Characterization of *Salmonella* Isolates From Pigs

**FIGURE 1:**
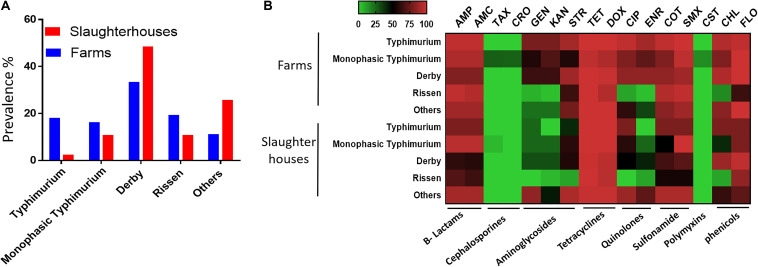
The serovar and antibiotic resistance diversity of *Salmonella* across farm and slaughterhouses. **(A)** Spatial variation of the distribution of multiple *Salmonella* serovars isolated from fecal samples analyzed in this study. **(B)** Distribution of the average resistance (in percent) of various serovars regardless of the source of isolation toward multiple antibiotics. The names of the antibiotics are abbreviated as ampicillin (AMP), amoxicillin (AMC), cefotaxime (TAX), ceftriaxone (CRO), gentamicin (GEN), kanamycin (KAN), streptomycin (STR), tetracycline (TET), doxycycline (DOX), ciprofloxacin (CIP), enrofloxacin (ENR), sulfa-trimethoprim (COT), sulfamethoxazole (SMX), colistin (CST), chloramphenicol (CHL), and florfenicol (FLO).

A total of 337 *Salmonella* strains were isolated from 1732 fecal samples of healthy finishing pigs randomly selected from 45 pig farms and two slaughterhouses in Henan province in China between March 2017 and November 2017. *S.* Derby was the most abundant serovar in both farms and slaughterhouses (Figure 1A). The prevalence of *S.* Typhimurium and monophasic variant serovars in the farms was higher than that in slaughterhouses. We also noticed that the isolation rate of the monophasic *S.* Typhimurium serovar was higher than other *S.* Typhimurium in slaughterhouses (Figure 1A). Most of the isolates exhibited multidrug resistance (resistant to three or more different antimicrobials classes) pattern by showing resistance to β-lactams, sulfonamides, phenicols, and tetracycline classes. Notably, the monophasic Typhimurium isolates (*N* = 7) from finishing pigs showed the highest resistance to all examined antimicrobials (Figure 1B). Importantly, four monophasic *S.* Typhimurium isolates (*Sal*_13.3, *Sal*_13.5, *Sal*_136, and *Sal*_15.5) obtained from pig farms showed resistance to colistin (MIC = 4 mg/L). These four isolates were isolated in 2017; three of them (*Sal*_13.3, *Sal*_13.5, and *Sal*_13.6) were obtained from the same farm located in Jiyuan city; however, *Sal*_15.5 was obtained from another farm located in Changhui city.

Polymerase chain reaction screening of all isolates also confirmed that only these four isolates harbored the *mcr-1* gene. No positive isolates were identified from slaughterhouses. MLST subtyping and *in silico* serotyping, including the serum agglutination test and PCR assay, confirmed that the four strains were *Salmonella enterica* serovar 4,[5],12:i- and belonged to sequence type 34 (ST34) (Table 1).

**TABLE 1:** Antimicrobial susceptibility, conjugation rate and whole genome analysis of mcr-1 positive strains isolated in this study.

	Antibiotic classes	Antibiotics	*Sal*_13.3		*Sal*_13.5		*Sal*_13.6		*Sal*_15.5	
						
			MIC (mg/L)	Related genes	MIC (mg/L)	Related genes	MIC (mg/L)	Related genes	MIC (mg/L)	Related genes
**Antimicrobial**	**β-Lactam and**	Ampicillin	>128	*bla*_*OXA–*__1_	>128	*bla*_*OXA–*__1_	>128	*bla*_*OXA–*__1_	>128	*bla*_*OXA–*__1_
	**β-lactam inhibitor**	Amoxicillin Clavulanic	>64/32		>64/32		>64/32	*bla*_*TEM–*__1__*B*_	>64/32	*bla*_*TEM–*__1__*B*_
**susceptibility testing**	**Aminoglycoside**	Kanamycin	>128	*aph(3’)-Ia, aadA2, aac(6’)-Ib-cr, aadA1, aph*(4)*-Ia, aac(6’)-Iaa, aac*(3)*-IV, aph(3‘’)-Ia*	>128	*aadA1, aadA2, aac(6’)-Ib-cr, aph*(4)*-Ia, aac(6’)-Iaa, aac*(3)*-IV, aph(3‘’)-Ia*	>128	*aph(3’)-Ia, aadA2, aac(6’)-Ib-cr, aph*(4)*-Ia, aac(6’)-Iaa, aac*(3)*-IV, aph(3”)-Ib, aph*(6)*-Id*	>128	*aph(3’)-Ia, aadA2, aac(6’)-Ib-cr, aadA1, aph*(4)*-Ia, aac(6’)-Iaa, aac*(3)*-IV, aph(3”)-Ib, aph*(6)*-Id*
		Streptomycin	128		128		128		128	
		Gentamicin	32		16		16		32	
	**Polymyxins**	Colistin	4	*mcr-1*	4	*mcr-1*	4	*mcr-1*	4	*mcr-1*
	**Fluoroquinolone**	Ciprofloxacin	4	*oqxB, oqxA*	4	*oqxB, oqxA*	4	*oqxB, oqxA*	4	*oqxB, oqxA*
	**Phenicol**	Chloramphenicol	128	*cmlA1, catB3*	128	*catB3*	128	*cmlA1, catB3*	128	*cmlA1, catB3*
		Florfenicol	>128	*floR, arr-3*	>128	*floR, arr-3*	64	*arr-3*	64	*arr-3*
	**Sulfonamide**	Sulfaxisazole	>512	*sul3, sul2, sul1*	256	*sul2, sul1*	>512	*sul3, sul2, sul1*	>512	*sul3, sul2, sul1*
	**Trimethoprim/sulfonamide**	Trimethoprim Sulfamethoxazole	>32/608	*dfrA12, sul3, sul2, sul1*	>32/608	*dfrA12, sul2, sul1*	>32/608	*dfrA12, sul3, sul2, sul1*	>32/608	*dfrA12, sul3, sul2, sul1*
	**Tetracyclines**	Tetracycline	128	*tet*(A)	128	*tet*(A)	128	*tet*(A)	128	*tet*(A)
		Doxycycline	32		32		32		64	
	**Cephalosporines**	Ceftriaxone	<0.125		<0.125		<0.125		<0.125	
		Cefotaxime	<0.125		<0.125		<0.125		<0.125	
**Collection time**			2017		2017		2017		2017	
**Sequence type**			ST34		ST34		ST34		ST34	
***mcr-1* location**			IncHI2 plasmid		IncHI2 plasmid		IncHI2 plasmid		Chromosome	
**Conjugation rate**			2 × 10^–3^		1 × 10^–4^		1 × 10^–3^		Failed	

### Bacterial Conjugation Assay

The *mcr-1* gene in *Sal*_13.3, *Sal*_13.5, and *Sal*_13.6 strains could be transmitted to *E. coli* J53 (streptomycin- and rifampicin-resistant) with a conjugation efficiency of 2 × 10^–3^, 1 × 10^–4^, and 1 × 10^–3^ per donor cell, respectively. However, conjugal transfer failed in *Sal*_15.5.

### Antimicrobial Susceptibility and Related Genetic Determinants

**FIGURE 2:**
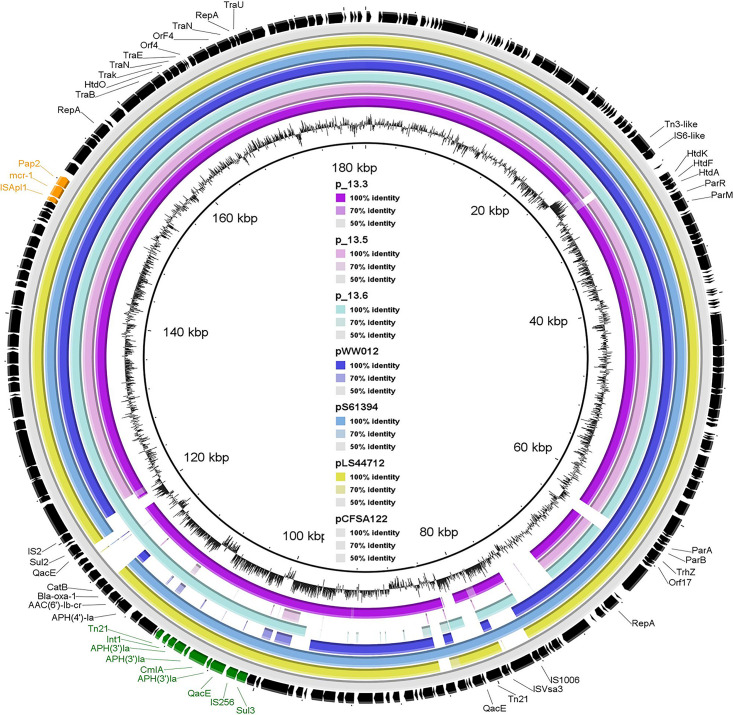
Sequence comparison of reconstructed *mcr-1*-positive plasmid from whole-genome sequence of *SAL*_13.3, *SAL*_13.5, *SAL*_13.6 with pLS44712 (NZ_CP035918), pS61394 (NZ_CP035916), pWW012 (NZ_CP022169), and pCFSA122-1 (NZ_CP033224.2) from a Chinese pork sample as a reference strain. Black arrows refer to pCFSA122-1, purple color refers to p*SAL*_13.3, pink color refers to p*SAL*_13.5, light blue color refers to *SAL*_13.6, dark blue color refers to pLS44712, blue color refers to pS61394, and yellow color refers to pWW012.

All four *mcr*-carrying isolates showed multidrug resistance pattern but were sensitive to carbapenems and cephalosporines (Table 1). Phenotypic results correlated with the presence of the different antimicrobial resistance genes. As illustrated in Table 1, the four *mcr-1-*positive isolates exhibited MIC to colistin = 4 mg/L. They were phenotypically resistant to tetracyclines, which was confirmed by the presence of *tet*(A) gene. They also showed resistance to ampicillin and amoxicillin–clavulanic acid conferred by *bla*_*OX*__*A*__–__1_ or *bla*_*TEM–*__1__*B*_ (Table 1). Furthermore, co-resistance was observed for streptomycin, kanamycin, and gentamicin with an average of six different genes, which were responsible for aminoglycoside resistance. *aac(6’)Ib-cr* gene, responsible for resistance to the aminoglycosides in addition to quinolones, was also detected in all isolates. *Sal*_13.3 and *Sal*_13.5 were resistant to florfenicol (MIC > 128 mg/L) due to the presence of *flor* and *arr-3* genes. However, *Sal_*13.6 and *Sal_*15.5 exhibited higher MIC value (64) and that may be due to the presence of the *flor* gene, which was specifically presented in these two isolates. All isolates showed resistance to chloramphenicol, which is encoded by *cmlA1 and catB3*, and ciprofloxacin, which is encoded by *oqxB* and *oqxA.* Importantly, *mcr-1*, along with one copy of Class-1 integron, has been identified in p*SAL*_13.3 and p*SAL*_13.6. Class-1 integron in both isolates have three copies of *aph*(4)*-Ia* and one copy of *cmlA* in their gene cassettes (Figure 2).

### Genetic Background of the *mcr-1* Gene

**FIGURE 3:**
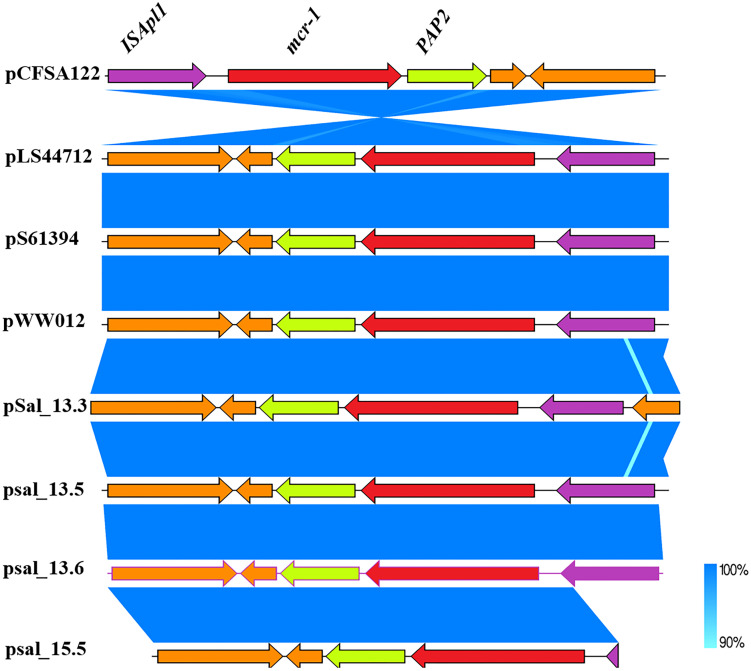
Genetic comparison of *mcr-1* gene context between *SAL*_13.3, *SAL*_13.5, *SAL*_13.6 with pLS44712 (NZ_CP035918), pS61394 (NZ_CP035916), pWW012 (NZ_CP022169), and pCFSA122-1 (NZ_CP033224.2). N.B. Blue color in *mcr-1* gene context refers to 90–100% identity and *SAL*_15.5 carries *mcr-1* gene on chromosome.

The *mcr-1*-coding sequence was located directly upstream of an open reading frame encoding *PAP*2 family protein, which is frequently associated with *mcr-1.* The complete version of *ISApl1* located downstream of the *mcr-1* cassette was detected in *SAL*_13.3, *SAL*_13.5, and *SAL*_13.6, and an incomplete version of *ISApl1* was detected in *SAL*_15.5 (Figure 3).

### The Plasmids Carrying *mcr-1* Gene

The p*SAL*_13.3_*mcr-1* (175,265 bp) (Supplementary Figure S1), p*SAL*_13.5_*mcr-1* (163,786 bp) (Supplementary Figure S2), and p*SAL*_13.6_*mcr-1* (171,333 bp) (Supplementary Figure S3) are IncHI2 plasmid, reconstructed from the whole genomic sequence by using PLANCETw. All *mcr*- plasmids were successfully transferred by conjugation to the recipient strain, signifying their ability to carry the *mcr* genes between isolates. For all isolates, genome sequencing allowed the detection of *mcr*-1 genes in the same contigs as replicons of the plasmid families. The plasmid sequence had a typical IncHI2-type backbone coding replication and conjugative transfer region, and also carried IncF replicons. In general, the structure of the IncHI2 plasmids seemed to present high similarity, with all of them having the conjugative transfer system, HigB-HigA toxin–antitoxin system for plasmid maintenance, and a tellurium resistant operon (Supplementary Figures S1–S3). Additionally, these plasmids also harbored antimicrobial resistance genes of different categories, including aminoglycosides, beta-lactams, tetracycline, phenicols, sulfonamides, and trimethoprim (Figure 2 and Supplementary Figure S4). They also showed >95% nucleotide sequence identity to the corresponding region of the *mcr-1* positive InCHI2 plasmids obtained from Chinese *S. enterica* isolates pLS44712 (NZ_CP035918), pS61394 (NZ_CP035916), pWW012 (NZ_CP022169), and pCFSA122-1 (NZ_CP033224.2) (Figure 2). The plasmids harbored *mcr-1* along with many other resistance genes.

### Comparative Genomics and Clonal Nature of Chinese *mcr*-Positive Isolates

**FIGURE 4:**
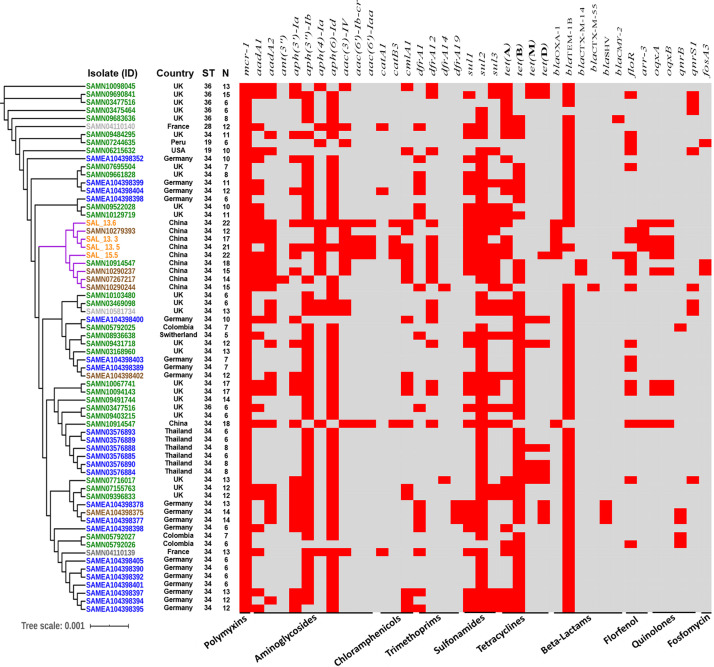
Global phylogenomic analysis of *mcr-1*-positive *S*. Typhimurium and monophasic isolates from different hosts retrieved from NCBI database (sample ID). Green color refers to human strains, blue color refers to animal strains, brown color refers to pork strains, gray color refers to environmental strains, and orange color refers to the isolated strains in this study. N refers to the numbers of resistant genes. ST refers to the sequence type.

In order to evaluate the relationship between the four *mcr*-carrying isolates and all *mcr-1* positive *S.* Typhimurium and monophasic variant isolates from different countries and sources, the phylogenetic tree of 62 *mcr*-*1* positive isolates, including 34 monophasic *S.* Typhimurium and 28 *S.* Typhimurium isolates with genomes available in the NCBI database, was used to test the clonal feature (Figure 4). Except for SAMN10914547, all Chinese, including pig, pork, and human isolates, were clustered together, composed of two closely related independent subclades, and the whole-genome sequencing of the pork and human isolates showed that they were monophasic *S.* Typhimurium or 4,[5],12:i:-, also belonging to ST34, and all of them, except SAMN10290237, have the *mcr-1* gene carried on IncHI2 plasmids. Additionally, Pan-genome analysis with Roary pipeline tool (Page et al., 2015) exhibited similar patterns of the genomes of all Chinese isolates. Further analysis revealed that 4597 (85.5%) out of 5663 genes were conserved among the completed genomes of all nine Chinese isolates (Figure 4 and Supplementary Figure S5). These results suggest the vital role of the food chain in the dissemination of *mcr-*carrying *S.* Typhimurium in China. We also found that there is a small difference in distance between phylogenetic branches of all *mcr-1*-positive InCHI2 plasmids obtained from Chinese *S. enterica* isolates pLS44712 (NZ_CP035918), pS61394 (NZ_CP035916), pWW012 (NZ_CP022169), and pCFSA122-1 (NZ_CP033224.2), indicating the close relation among these plasmids, which are from the same Inc type (Supplementary Figure S6). *Salmonella* isolate CFSA12 was reported as a mutant strain that has lost the *mcr-1* gene from its wild strain WW012 of serovar Typhimurium (Hu et al., 2019).

### Global Phylogenomic Analysis of *mcr-1-*Carrying *S.* Typhimurium

We retrieved all *mcr-1-*carrying *Salmonella* Typhimurium from the NCBI database in addition to the isolates obtained in this study to construct the phylogenomic tree (Figure 4). We noticed that *mcr-1-*carrying *S.* Typhimurium has been isolated from various sources like humans, animals, food products, and the environment. A small difference in distance between phylogenetic branches of the isolates was identified with a scale bar at 0.01, indicating a very close genetic relationship between the isolates obtained from different sources. The whole-genome analysis of all *mcr-1-*carrying isolates exhibited 58 of the 66 isolates, including those isolates reported in this study (Figure 4 and Supplementary Figure S7). These results highlighted an interesting host preference of *mcr-1* gene and a worldwide prevalence of the *mcr*-positive *Salmonella* Typhimurium ST34. All isolates including the strains determined in this study carry antimicrobial resistance genes for aminoglycosides and β-lactams. *aph*(6)*-Id* (55 isolates, 83%) and *bla*_*TEM–*__1__*B*_ (58 isolates, 87.8%) were the most detected genes among all the *mcr-1-*carrying isolates. *Bla*_*OXA–*__1_ gene was only detected in Chinese isolates. We noticed that among all isolates, 47 (71%) were isolated between 2016 and 2018 (Supplementary Figure S7). Interestingly, the Chinese isolates have the highest number of antimicrobial resistance genes among all the examined isolates (Figure 3).

## Discussion

Monophasic *Salmonella* 4,[5],12:i:- has become a global new epidemic multidrug-resistant clone associated with animal and human infections (Hopkins et al., 2010). For unknown reason, this particular clone was preferentially associated with swine, particularly in finishing herds, where the spillage of the intestinal contents during slaughter is a primary risk factor for the cross-contamination (Rodríguez and Suárez, 2014; Paudyal and Yue, 2019). As mentioned earlier, *S. enterica* 4,[5],12:i:- ST34 carrying *mcr-1* have been reported in humans and pork (Li et al., 2016; Alicia et al., 2018). Here, *S. enterica* 4,[5],12:i:- isolated from asymptomatic finishing pigs were used to evaluate their role in *mcr-1* gene transmission via the food chain.

We have investigated the prevalence of *mcr-1* gene among *Salmonella* strains obtained from pigs in farms in Henan, China. Out of 337 *Salmonella* isolates, four (1.1%) isolates were positive for *mcr-1*. All other *mcr* genes (*mcr-2* to *mcr-8*) have not been detected by the established multiplex PCR assay (Wang et al., 2018). The incidence rate of *mcr-1* in *Salmonella* was much lower as compared to other Enterobacteriaceae (Liu et al., 2016; Malhotra-Kumar et al., 2016). The low prevalence of *Salmonella* harboring *mcr-1* was also reported in other studies in China, England, and Wales (Doumith et al., 2016; Li et al., 2016).

All *mcr-1*-positive *S. enterica* 4,[5],12:i:- strains, belonging to ST34, were often related to an evolving multidrug-resistant *S*. *enterica* 4,[5],12:i:- clade in Australia, China, Italy, and United States (Li et al., 2016; Alicia et al., 2018; Elnekave et al., 2018). It is likely that the clonal dissemination of *S*. *enterica* 4,[5],12:i:- ST34 contributes to the spread of the *mcr-1* gene among food animals in China (Lu et al., 2019) and may become a global significant public health concern (Elnekave et al., 2018; Mather et al., 2018; Monte et al., 2019).

It has been noticed that *mcr-1* was carried also on IncHI2 plasmids in three of our strains, which is similar to that reported for *S.* Typhimurium ST34 (Linxian et al., 2017). Conjugation experiments confirmed the ability of all the isolates except *SAL_*15.5 to mobilize the antimicrobial-resistant gene to a recipient strain, and Genome sequencing data verified the presence of the conjugative determents. IncHI2/HI2A plasmids are typically large (García-Fernández and Carattoli, 2010), multidrug-resistant plasmids that have been accompanied by a range of antimicrobial and metal resistance genes in *Salmonella* species from humans and food-producing animals (Linxian et al., 2017; Elnekave et al., 2018; Mather et al., 2018; Paudyal et al., 2018; Biswas et al., 2019; Elbediwi et al., 2019; Lu et al., 2019; Monte et al., 2019). The presence of IncHI2 plasmids in *Salmonella* serovars indicates that horizontal transfer of *mcr-1*-harboring plasmids might have also contributed to the spread of *mcr-1* and other resistant determinants in these bacteria (Liu et al., 2018). IncHI2 plasmids also carried a diversity of antimicrobial resistance genes from different categories, including aminoglycosides, beta-lactams, tetracycline, sulfonamides, and phenicols. Common antimicrobials were used to be administered during the rearing cycle in pig production and could persist for a long period in food-producing animals. These plasmids also contained genes encoding small multidrug resistance efflux transporter (QacE) conferring resistance to quaternary ammonium compounds (QACs). QAC has been commonly used as disinfectants with a wide application in the food industry (Quinn et al., 2015). Resistance to disinfectants presumably confers these clones the capacity to select and survive under available extreme conditions.

The *mcr-1* flanking regions have also been reported in previous studies (Li et al., 2016; Malhotra-Kumar et al., 2016; Linxian et al., 2017). The *ISApl1* flanking *mcr-1* gene seems to play a crucial role in the dissemination of *mcr-1* transposition between various incompatibility families of plasmids (Snesrud et al., 2016; Poirel et al., 2017), particularly in IncHI2 plasmids (Li et al., 2016; Linxian et al., 2017). In this study, we detected complete and incomplete copies of *ISApl1* element downstream the *mcr-1* gene, fixing the *mcr-1* gene to the plasmid. These differences in the surrounding regions of *mcr-1* probably indicate different stages in the evolution of the plasmid (Snesrud et al., 2016) or due to inadequate sequencing depth and coverage.

*Salmonella* serovars have a wide host range and can be transmitted to a broad diversity of animals, including mammals, birds, fish, and insects (Pan et al., 2019). Besides, *Salmonella* can grow in plants and can survive in protozoa, soil, and water (Silva et al., 2014; Pan et al., 2018). Hence, broad-host-range *Salmonella* can be transmitted via feces from wild animals, farm animals, and pets or by consumption of a wide variety of common foods: poultry, beef, pork, seafood, milk, fruit, and vegetables (Pan et al., 2019; Elbediwi et al., 2020). Phylogenomic analysis of four strains, determined in this study, with all available *mcr-1*-carrying *S.* Typhimurium and monophasic isolates from swine, poultry, humans, and environment, showed that these four strains were closely related and clustered together with four additional Chinese pork isolates and one human isolate (Figure 4). However, *in silico* serotyping of these isolates were monophasic *S.* Typhimurim (4,[5],12:i:-), and besides sharing the same sequence type (ST34), all, except for SAMN10290237, have the *mcr-1* gene carried on IncHI2 plasmids. In addition, there is very limited genetic difference in the distance between the branches of the evolutionary tree of the genomes, indicating the consistency with the sequence type results. We could not prove the potential role of the transmission by performing *in vivo* experiments. These findings suggested that pork, pigs, and human monophasic *S.* Typhimurim (4,[5],12:i:-) isolates might be from the same source, and pork-borne transmission played a crucial role in the transmission of *mcr-1-*carrying *S. enterica* 4,[5],12:i:- ST34. Further enhanced surveillance should pay particular attention to the IncHI2-mediated *mcr-1* transmission in monophasic *S. enterica* ST34.

Notably, the closely related Chinese swine-originated isolates were reported from Henan and Guangxi provinces (Supplementary Figure S4), top pig producers in China with the density exceeding 100 hogs per 100 acres (USDA, 2009; Gale et al., 2012). Additionally, Yang et al. (2019) reported that these two provinces were the highest antibiotics-consuming hot spots of pig production in China.

## Conclusion

This study provided essential knowledge of the pig–pork chain in the transmission of *mcr-1* by *Salmonella* 4,[5],12:i:- in China. In addition, it highlighted the importance of the occurrence of IncHI2 plasmids in *S. enterica* 4,[5],12:i:-, which may act as a vehicle for the *mcr-1* gene and multiple antimicrobial-resistant genes during their dissemination through the food chain. Furthermore, the spread of similar IncHI2-like plasmids and *Salmonella* serovar 4,[5],12:i:- clones carrying *mcr-1* emphasizes the requirements for internationally coordinated response strategies and continuing surveillance to mitigate *mcr*-carrying bacteria dissemination.

## Data Availability Statement

The datasets generated for this study can be found in the NCBI Bioproject, accession number PRJNA573539.

## Ethics Statement

No ethical approval was required for the current study. Fecal samples were obtained from farm pigs, with the permission of the farmers. Live animals were not handled directly. Oral agreement and permission was obtained from the farmers as well as the slaughterhouse manager before the sampling.

## Author Contributions

ME designed the study and prepared the first draft, figures, and tables. HP, ME, and BW did the data analysis. ZJ collected the samples and did the microbiological isolation. SB and YL reviewed the manuscript. MY and ME finalized the manuscript and managed the project. All authors contributed to the article and approved the submitted version.

## Conflict of Interest

The authors declare that the research was conducted in the absence of any commercial or financial relationships that could be construed as a potential conflict of interest.

## Footnotes

1 ftp://ftp.ncbi.nlm.nih.gov/blast/db/FASTA/nr.gz

2 https://cge.cbs.dtu.dk/services/

3 http://blast.ncbi.nlm.nih.gov/Blast.cgi

4 https://www.snapgene.com/

5 http://phylobench.vital-it.ch/raxml-bb/index.php

## Supplementary Material

FIGURE S1 Genetic structure of *mcr-1-carrying* p*Sal*_13.3.

FIGURE S2 Genetic structure of *mcr-1-*carrying p*Sal*_13.5.

FIGURE S3 Genetic structure of *mcr-1-*carrying p*Sal*_13.6.

FIGURE S4 Sequence comparison of reconstructed mcr-1-positive plasmid from whole-genome sequence of SAL_13.3, SAL_13.5, and SAL_13.6 with highlighting the presence of antimicrobial resistance genes.

FIGURE S5 Phylogenomic analysis, metadata, and comparative genomics analysis of Chinese *mcr-1*-positive monophasic *S*. Typhimurium (4,[5],12:i:-) in addition to *mcr-1* location in these isolates. Green color refers to human isolates, brown color refers to pork, and orange color refers to the isolated strains in this study. ^∗^*Salmonella* isolate CFSA12 was reported as a mutant strain that has lost the *mcr-1* gene from its wild strain *Salmonella* Typhimurium WW012.

FIGURE S6 Phylogenomic analysis of reconstructed mcr-1-positive plasmid from whole-genome sequence of SAL_13.3, SAL_13.5, and SAL_13.6 genome sequence of *SAL*_13.3, *SAL*_13.5, *SAL*_13.6 with pLS44712 (NZ_CP035918), pS61394 (NZ_CP035916), pWW012 (NZ_CP022169), and pCFSA122-1 (NZ_CP033224.2) from a Chinese pork sample as a reference strain.

FIGURE S7 Circular comparison and phylogenomic analysis a of global *mcr-1*-positive *S*. Typhimurium and monophasic variant according to year and source of isolation, sequence type, and place of isolation. Ring 1 refers to year of isolation, ring 2 refers to sequence type, ring 3 refers to isolation sources, and ring 4 refers to the place of isolation.
