# Corrigendum: Genomic Characterization of *mcr-1-*carrying *Salmonella enterica* Serovar 4,[5],12:i:- ST 34 Clone Isolated From Pigs in China

**DOI:** 10.3389/fbioe.2020.00842

**Published:** 2020-08-06

**Authors:** Mohammed Elbediwi, Beibei Wu, Hang Pan, Zenghai Jiang, Silpak Biswas, Yan Li, Min Yue

**Affiliations:** ^1^Institute of Preventive Veterinary Sciences, Department of Veterinary Medicine, College of Animal Sciences, Zhejiang University, Hangzhou, China; ^2^Zhejiang Province Center for Disease Control and Prevention, Hangzhou, China; ^3^College of Veterinary Medicine, Henan University of Animal Husbandry and Economy, Zhengzhou, China; ^4^Zhejiang Provincial Key Laboratory of Preventive Veterinary Medicine, College of Animal Sciences, Zhejiang University, Hangzhou, China

**Keywords:** *Salmonella enterica* serovar 4, [5], 12:i:-, ST34, Colistin, *mcr-1*, food chain

Beibei Wu was not included as an author in the published article. The corrected Author Contributions Statement appears below.

ME designed the study and prepared the first draft, figures, and tables. HP, ME, and BW did the data analysis. ZJ collected the samples and did the microbiological isolation. SB and YL reviewed the manuscript. MY and ME finalized the manuscript and managed the project. All authors contributed to the article and approved the submitted version.

The authors apologize for this error and state that this does not change the scientific conclusions of the article in any way. The original article has been updated.

