# Supplementary figures and images for: Genomic Characterization of mcr-1-carrying Salmonella enterica Serovar 4,[5],12:i:- ST 34 Clone Isolated From Pigs in China
Source: Front Bioeng Biotechnol. 2020 Jun 30;8:663. doi: 10.3389/fbioe.2020.00663 (PMC7344297; doi:10.3389/fbioe.2020.00663)

# Supp. Figure 1

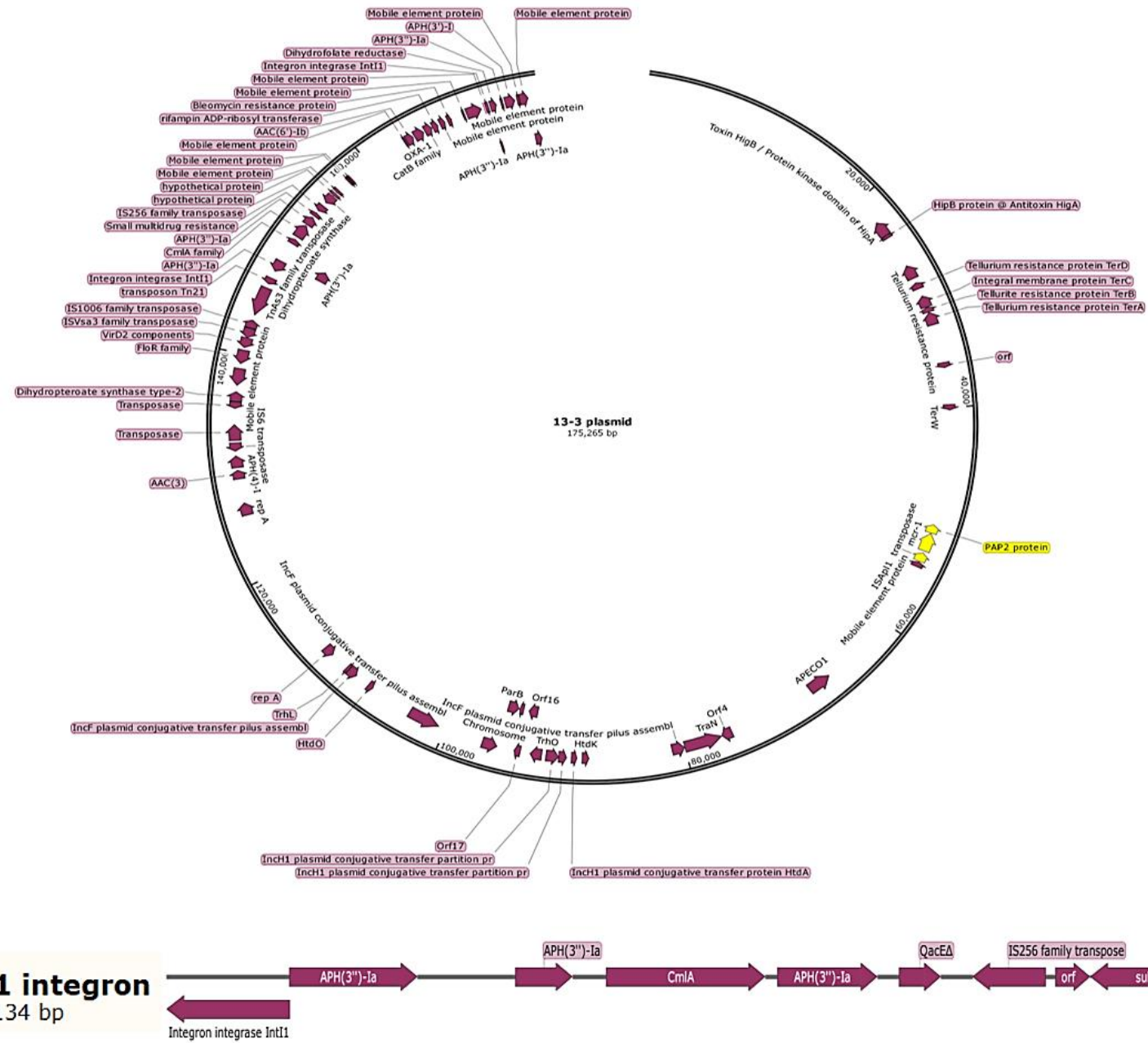

Supp. Figure 2

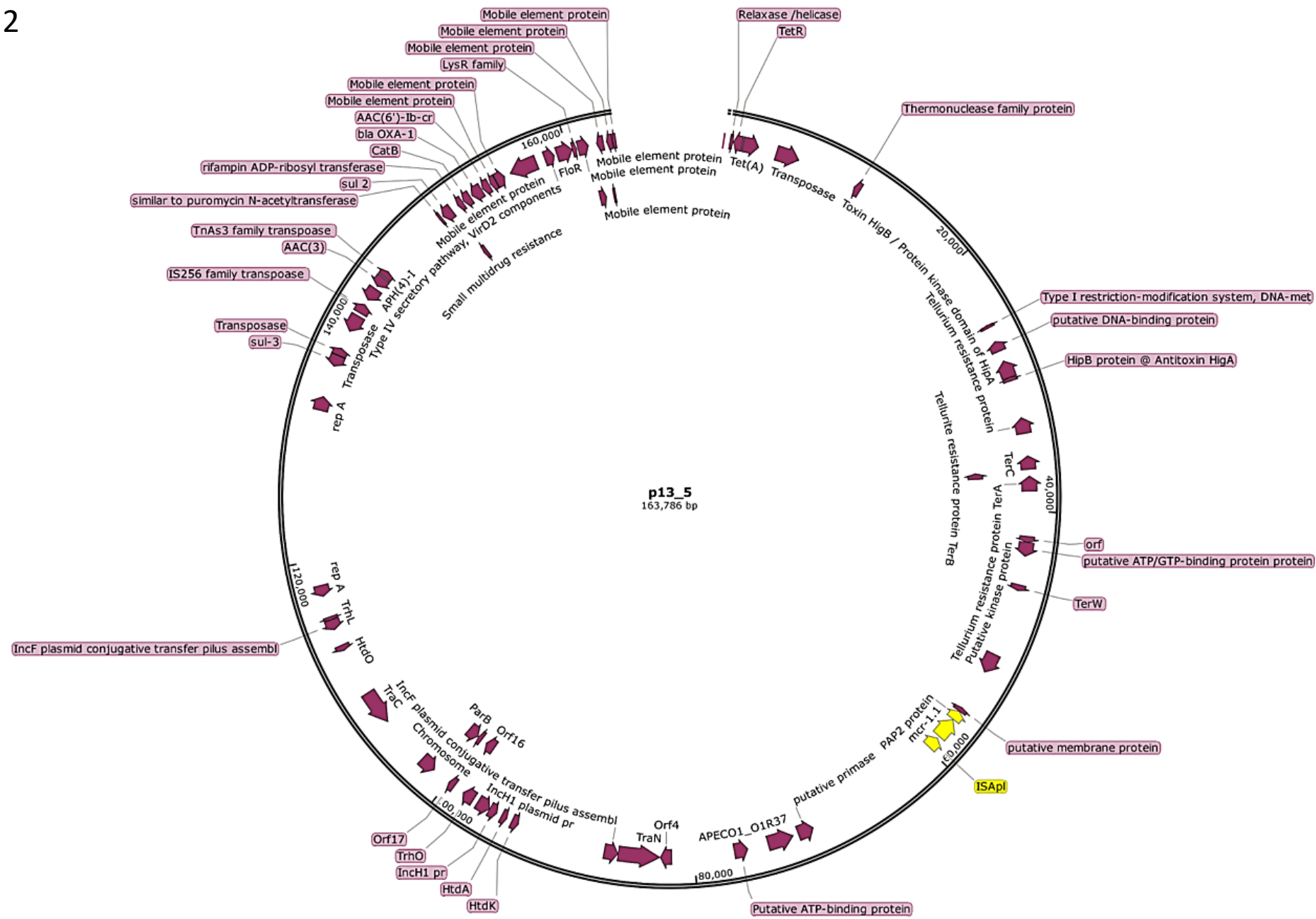

## Supp. Figure 3

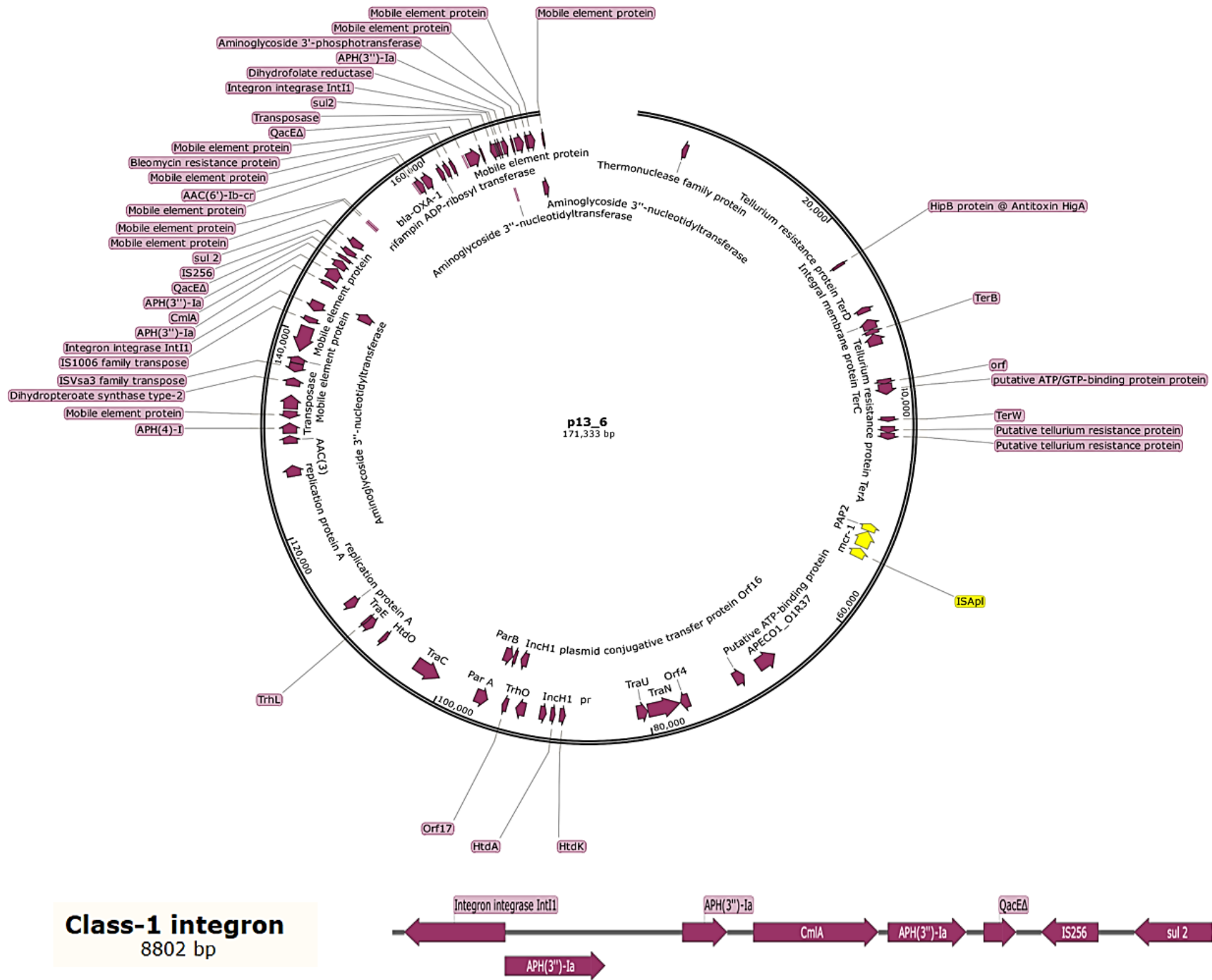

Supp. Figure 4

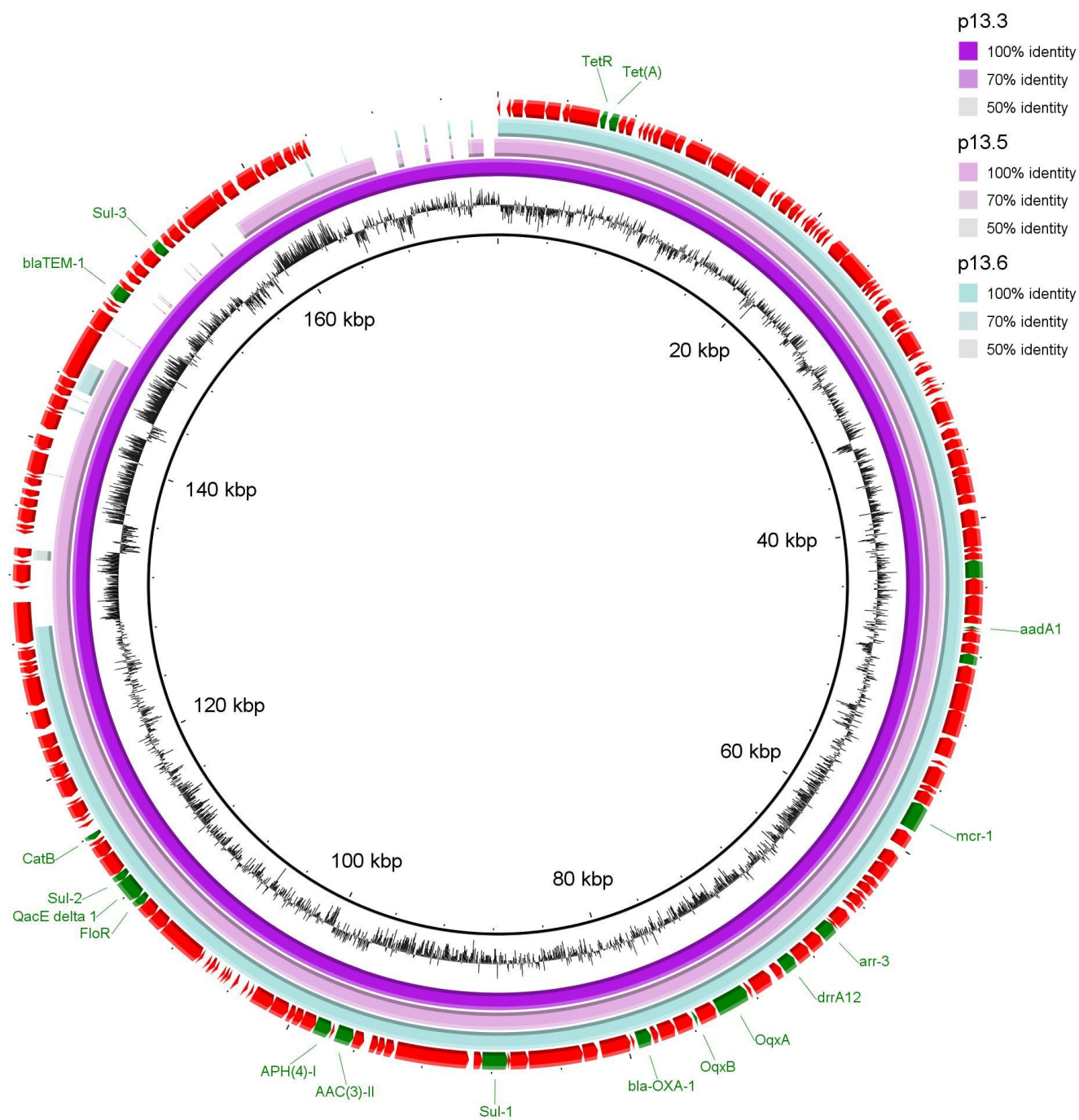

Supp. Figure 5

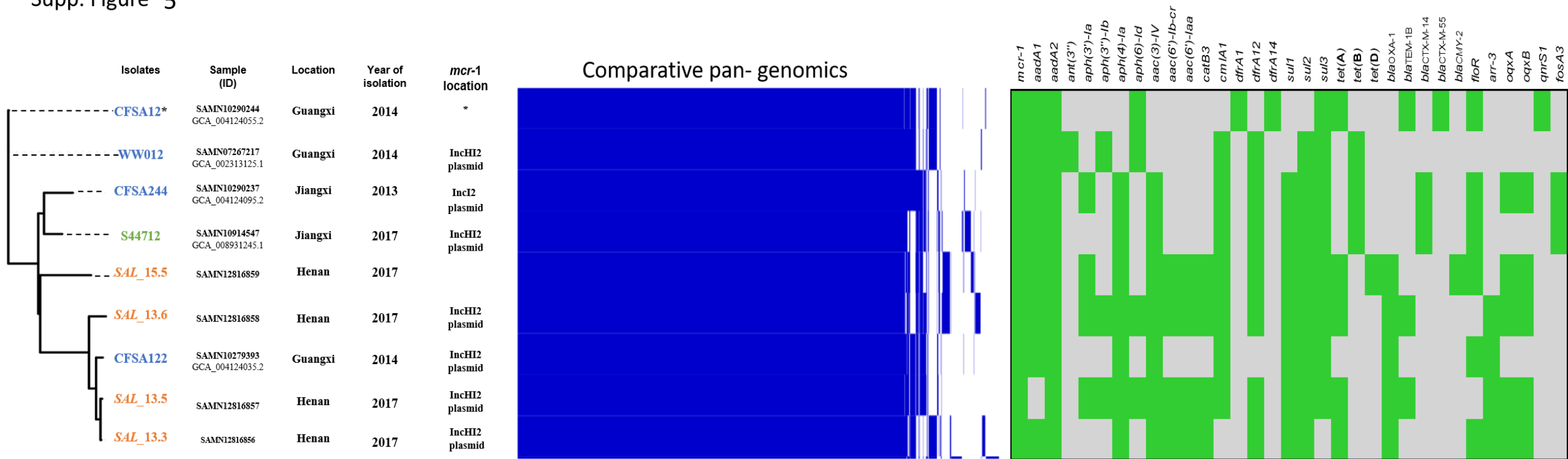

Supp. Figure 6

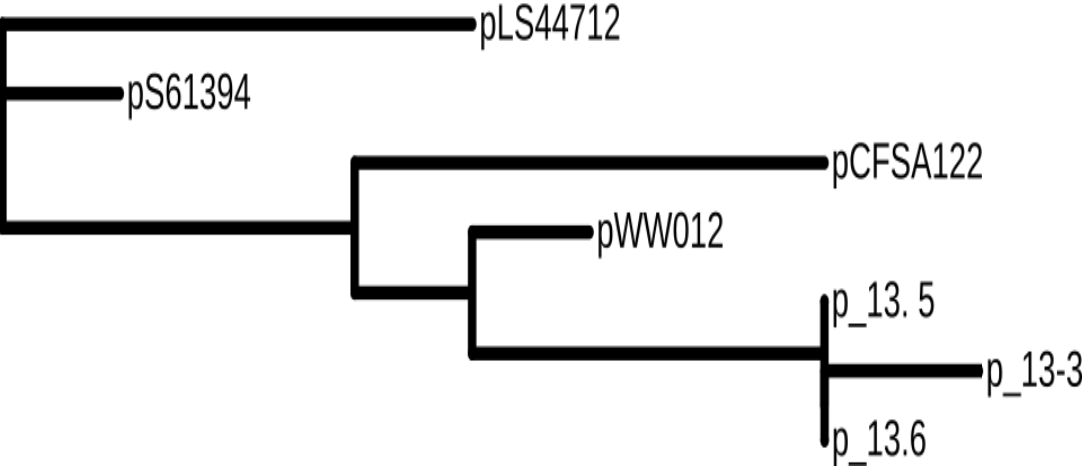

Tree scale: 0.000001

Supp. Figure 7

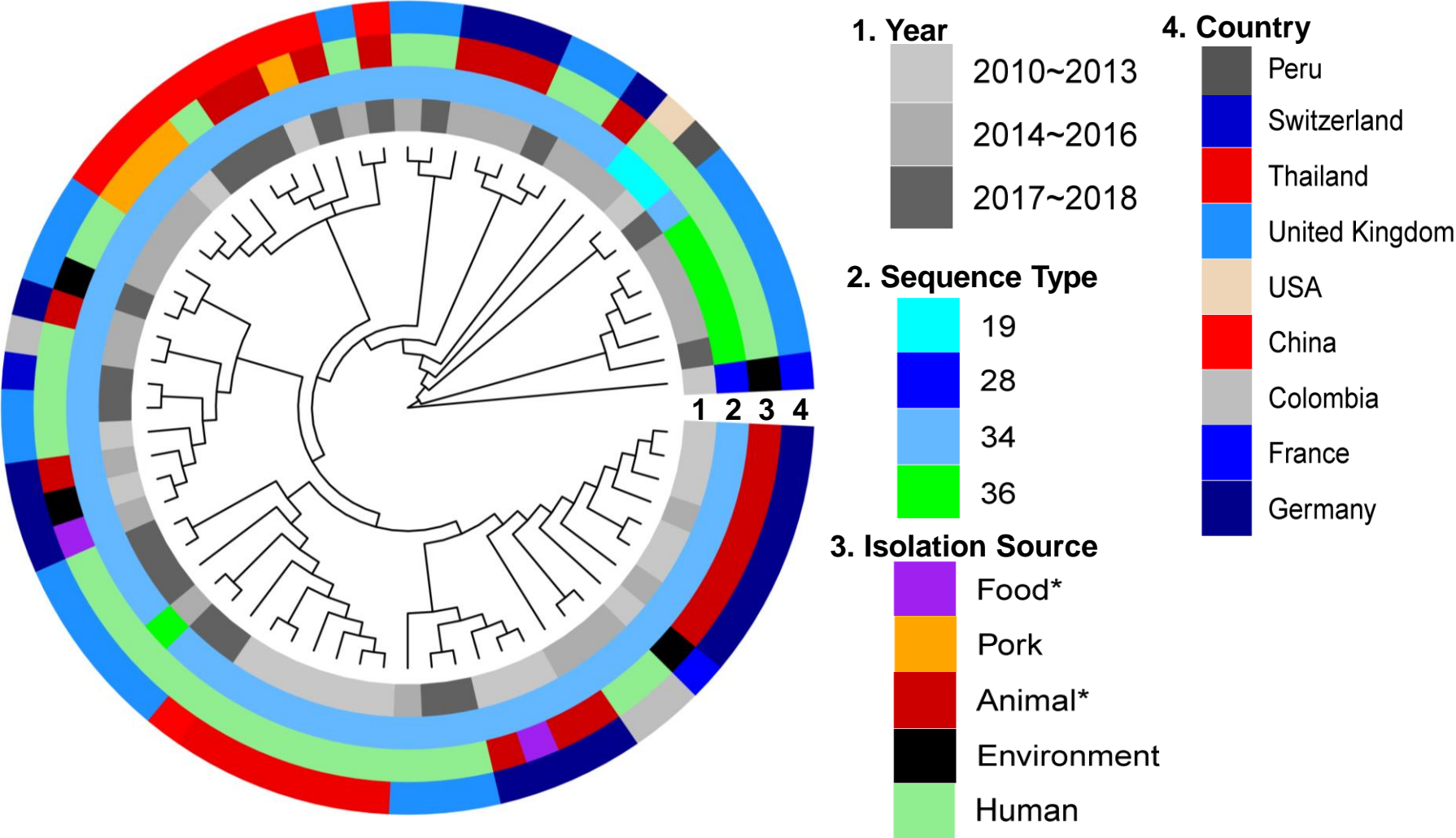

Supplement: FIGURE S1 — Genetic structure of mcr-1-carrying pSal_13.3. [file Image_1.pdf]
